# Enhancing the adsorption capacity of organic and inorganic pollutants onto impregnated olive stone derived activated carbon

**DOI:** 10.1016/j.heliyon.2024.e32792

**Published:** 2024-06-12

**Authors:** Duygu Ova Ozcan, Mert Can Hendekci̇, Bikem Ovez

**Affiliations:** Chemical Engineering Department, Faculty of Engineering, Ege University, 35100, Bornova/Izmir, Turkey

**Keywords:** Olive stone waste, Impregnation, Adsorption modeling, Activated carbon, Response surface methodology

## Abstract

This study presents a sustainable approach to activated carbon production from olive stones in comparison to commercial ones, using various activating agents such as H_3_PO_4_, KOH, and ZnCl_2_, for enhancing the adsorption properties and versatile adsorption capability to remove a range of pollutants including copper ion, methylene blue, and 2,4-Dichlorophenoxyacetic acid from aqueous solutions. The performances of activated carbons across varying conditions such as pollutant concentrations, temperatures, pH levels, and adsorbent amounts were tested. Increased initial pollutant concentrations correlated with higher adsorption capacities. Maximum adsorption capacities were achieved at pH levels of 5, 10, and 2 for Cu, MB, and 2,4-D, respectively. For KOSAC, Cu removal rose from 27 % to 52 %, for ZOSAC, MB removal increased from 39 % to 65 %, and for ZOSAC, 2,4-D removal surged from 33 % to 99 % at varying adsorbent amounts. Model validation was carried out utilizing the kinetic models (PFO, PSO) and isotherm models (Langmuir, Redlich-Peterson). The PFO kinetic model and Langmuir isotherm model proved more suitability for Cu adsorption, whereas PFO and PSO kinetic models, along with Redlich-Peterson isotherm models, were more prominent for MB and 2,4-D adsorption. Thermodynamic analysis revealed that the adsorption of Cu and 2,4-D was exothermic, while MB adsorption was endothermic. By optimization of experimental conditions, the maximum adsorption capacities were attained at 30.34 °C and 297.65 mg L^−1^ for KOSAC-Cu, 48.62 °C and 269.37 mg L^−1^ for ZOSAC-MB, and 30.31 °C and 299.02 mg L^−1^ for ZOSAC-2,4-D sorption. This research highlights ZOSAC's potential as a cost-effective, eco-friendly solution for water treatment, contributing to environmental sustainability and economical feasibility.

## Highlights

• Impregnating olive stones with ZnCl_2_, H_3_PO_4_, KOH increased the sorption capacity.
• Presence of mesopores and –Cl group enhanced the adsorption of 2,4-D on ZOSAC.
• Maximum capacity was attained at 30.34 °C and 297.65 mg L^−1^ for KOSAC-Cu adsorption.
• PSO kinetic and Redlich-Peterson models were more prominent for MB adsorption.
• The adsorption of Cu and 2,4-D was exothermic, while MB adsorption was endothermic.

## 1 Introduction

The pollution of water sources is a significant environmental challenge confronting the contemporary world, resulting in an ecological imbalance that adversely affects the plant and animal life within the ecosystem. In recent times, the concern about the contamination of current water reservoirs with coloring agents, phenolic substances, nitrates, phosphates, pesticides, organic materials that can break down naturally, long-lasting organic substances, sediment, and ions of heavy metals have increasingly gained prominence [1]. The challenge escalates with rising consumption needs for drinking water driven by a growing population. Consequently, there is a worldwide call for the advancement and adoption of drinking water treatment technologies that are both more efficient and cost-effective [2]. Numerous methods exist for removing organic pollutants from drinking water, including photocatalytic degradation, ultrasound, biological oxidation combined with aerobic degradation, membrane electrodialysis, and ozonation. However, adsorption stands out among other methods for its versatility in removing both soluble and insoluble organic pollutants with high efficiency.

Carbon materials have diverse applications, including purifying water, air, and gas, treating waste, capturing carbon dioxide, supporting catalysts, and serving as electrochemical components. Nowadays, one prevalent application involves utilizing graphitic carbon nitride nanosheets for efficient photocathodic protection [3], alongside composites of TiO_2_ nanotubes [[4], [5], [6], [7], [8]]. Another well-known use of carbon is in water treatment through adsorption. The choice of adsorbent is a very important criterion for the development and effectiveness of a successful adsorption process. An effective adsorbent, whether synthetic or sourced from natural origins, should exhibit a strong adsorption interaction with targeted contaminants and possess significant removal capacity. Adsorbents are commonly derived from sources such as metal-organic frameworks, materials based on graphene, activated carbons, zeolites, clay minerals, biomass, carbon nanotubes, and polymeric materials. The use of cost-effective methods in the production of graphene-based adsorbents often results in the generation of low-quality materials in limited quantities. Moreover, graphene-based materials develop irremovable deposits and layer aggregation and have poor binding affinity for anionic molecules. These challenges reduce the efficiency of adsorbents based on graphene, limiting their potential applications. Although iron oxide nanomaterials prove to be effective adsorbents, their application in water treatment is acknowledged as hazardous. Zeolite adsorbents, despite exhibiting high capacities for adsorbing various pollutants, pose difficulties such as reduced mass and heat transfer, prolonged settling time, recycling difficulties, elevated pressure drop, and susceptibility to agglomeration at high dosages pose challenges. These factors collectively limit the broader applications of adsorption [9]. Considering all these disadvantages, a porous activated carbon showing a structurally well-defined surface area, that demonstrates robust mechanical stability, low specificity, rapid kinetics, and substantial adsorption capacity is still the most preferred type of adsorbent. However, commercially available activated carbon is considered costly because it originates from starting materials like coal that are both non-renewable and relatively costly thereby restricting its use as an adsorbent. This has sparked an increasing interest in generating activated carbon from affordable and renewable sources. The development of a diverse range of economical adsorbents from various waste materials, encompassing agricultural, industrial and municipal waste, is of particular environmental and circular economic importance because of their high efficiency, ease of synthesis, environmental friendliness, waste use and recycling to achieve cleaner production, particularly in applications like water treatment without toxic or carbon footprints, their sustainability and renewability [10].

In order to improve the efficiency and surface properties of adsorbents obtained from various biomass sources, waste materials are subjected to either physical or chemical activation. Physical activation avoids the incorporation of activating agent-derived impurities that may affect the chemical properties of the activated carbon positively but presents fewer advantages than its chemical counterpart. Specifically, chemical activation occurs at lower temperatures and results in high yields without producing burn-off char, reducing energy costs. It also yields a better-defined porous structure by causing micro and mesopores to develop in the nonporous precursor and thus increasing the surface area of the activated carbon. Activation agents widen the cellulosic structure of the precursor, support the activated framework during carbonization, and prevent the structure from shrinking. H_3_PO_4_ has become the most common chemical activating agent because it provides more pronounced porosity at lower environmental and toxicological constraints than ZnCl_2_ as well as a lower working temperature than KOH [11].

In this work, the organic and inorganic removal efficiencies of activated carbon derived from olive stones were enhanced by impregnating with H_3_PO_4_, ZnCl_2_ and KOH. Unlike previous approaches that often rely on a single type of activator, this study explores the capacity of diverse activators of different characteristics introducing variability and potentially enhanced adsorption properties. Secondly, this study addresses gaps in existing literature by conducting a comprehensive examination of various process parameters for olive stone based activated carbon production. By integrating kinetic, isotherm and thermodynamic modeling alongside optimization studies, it aims to provide a deeper understanding of adsorption performance and behavior as well as identifying optimal conditions for maximum adsorption efficiency. Thirdly, the practical relevance of the study is highlighted through the removal of three distinct groups of organic and inorganic contaminants (copper ion, methylene blue and 2,4-Dichlorophenoxyacetic acid) from aqueous solutions addressing a critical environmental concern and demonstrating the potential application of the synthesized activated carbon in real-world scenarios. By comparing the performance of waste-based activated carbon with commercially available alternatives, this research addresses a critical environmental concern and offers insight into the viability and competitiveness of the novel material. Importantly, this study aligns with principles of environmental sustainability by repurposing agricultural waste for activated carbon production, contributing to both water pollution mitigation and the eco-friendly utilization of agricultural waste. Thus, the unique and holistic approach of this research, from diverse activation methods to environmental relevance, fills critical gaps in the existing literature.

## 2 Materials and methods

### 2.1 Materials

In this investigation, olive stones supplied by the TARIS Cotton and Seed Agricultural Sales Cooperatives Union (Aydin, Turkey) served as the precursors for the production of activated carbons through chemical activation. The seeds underwent an initial washing with distilled water to eliminate dust, then dried at 110 °C for 24 h, and finally crushed to achieve the desired particle size using a Brook Crompton Series 2000 jaw crusher (UK). Chemical activators, namely zinc chloride (ZnCl_2_), phosphoric acid (H_3_PO_4_), and potassium hydroxide (KOH) (Merck), were employed in the activation process. To assess the performance of activated carbons derived from the olive stones (OSACs), commercial powdered activated carbon (PAC, Sigma-Aldrich) and granular activated carbon (GAC, Merck) were used as benchmarks. Contaminants such as copper (98 % purity, Cu(NO_3_)_2_•3H_2_O, Merck), 2,4-Dichlorophenoxyacetic acid (98 % purity, 2,4-D, Sigma-Aldrich), and methylene blue (MB, Merck) were utilized in the study. To enhance the solubility of 2,4-D in water, a specific amount of ethanol (Merck) and calcium chloride (CaCl_2_, Merck) was added. The stock solutions of all the reagents at a concentration of 1000 mg L^−1^ were prepared by dissolving them in distilled water with magnetic stirring. The pH of the solutions was adjusted using NaOH and HCl (Merck), and these chemicals were used without additional purification.

### 2.2 Preparation of chemically impregnated olive stone derived activated carbon

For the chemical activation, the pulverized stones underwent immersion in water-based solutions containing ZnCl_2_, H_3_PO_4_, and KOH, with an impregnation ratio (precursor/activator) of 1:1.5 (w/w). This soaking process occurred for approximately 2 h at 85 °C within a thermostatic shaker, followed by dehydration overnight at 110 °C in an oven. The carbonization of the samples treated with chemical activators took place at 600 °C for 2 h, employing a heating rate of 7 °C min^−1^ in a stainless steel vertical tubular reactor situated in a tubular furnace under a purified nitrogen flow (99.995 %, at a 25 mL min^−1^). The subsequent cooling process transpired under room conditions and a nitrogen gas atmosphere. At the conclusion of the activation period, the samples were brought to room temperature under nitrogen flow. The resulting activated carbon samples underwent washing with 0.1 M HCl in a Soxhlet extractor to dissolve and eliminate any residual ash and then were boiled with hot distilled water using a vacuum pump. To ensure the removal of any remaining Cl^−^ ions in the filtered samples, they underwent additional washing with AgNO_3_. This process continued until no precipitation or turbidity was observed, and the pH of the supernatant solution reached 6.5–7.0. Subsequently, all olive stone activated carbon (OSAC), as well as ZnCl_2_, H_3_PO_4_, and KOH impregnated activated carbon (ZOSAC, HOSAC, and KOSAC, respectively) samples, along with the commercial powdered activated carbon (PAC) and granular activated carbon (GAC), were oven-dried at 110 °C for 24 h, stored in desiccators, and sieved to the desired particle size.

### 2.3 Characterization of activated carbons

The elemental compositions of the ACs were determined by CHNS analysis (PerkinElmer 2400 CHNS/O Series II). The surface morphologies of the ACs were assessed using scanning electron microscopy (SEM) (Thermo Scientific Apreo S). X-ray Diffraction (XRD) analyses were performed to determine the crystal structure of the ACs (Rigaku Ultima IV). The surface area and porosity properties were determined using N_2_ adsorption-desorption at −196 °C (77 K) with a saturation pressure of 775 mmHg using an automated gas sorption system (Micromeritics, Model Gemini V). Before the adsorption measurements, individual samples were degassed at 573 K for 24 h at a final pressure of 0.02 mbar. The surface areas were calculated using the BET equation for relative pressures ranging between 0.05 and 0.35. In addition to the zeta potential analysis conducted to investigate the electrokinetic potential of the ACs (DLS – Particulate Systems, the pH point of zero charge (pH_PZC_) was determined by varying the initial pH (pHi) values from 2 to 10. The pH was adjusted using NaOH and HCl in a 1 M NaCl solution with 0.1 g of adsorbent subjected to 30 mL of the solution. Erlenmeyer flasks, each containing solutions with varied pH levels and the adsorbent, were subjected to agitation in a shaker at 25 °C until the solutions reached equilibrium. Subsequently, the activated carbon samples were filtered, and the solutions were analyzed using a digital pH meter to confirm the final pH (pHf) values of the aqueous solution.

### 2.4 Batch adsorption studies

The investigation assessed how the influence of contact time (1–18 h), initial contaminant concentration (10–300 mg L^−1^), temperature (30–50 °C), pH (2-10), and adsorbent dosage (0.01–0.1 g) affected the adsorption capabilities of the activated carbons (ACs). The sorptive capacities of the ACs were determined through batch experiments in 100-mL conical flasks with 50 mL of sorbate solutions at the specified initial concentration, temperature, and pH. These experiments took place in a thermostatic shaker operating at a constant speed of 90 rpm. Following a designated duration, the separation of the solution from the activated carbon was achieved through filtration using Macherey-Nagel MN 640 de filter paper. The control samples lacking activated carbon were employed, and each experiment was conducted in triplicate, with the obtained values subsequently averaged. The final concentrations of MB and 2,4-D were determined using UV spectroscopy (PG Instruments T80) at 665 and 228 nm, respectively, where the final concentration of Cu was measured by atomic absorption spectroscopy (Varian SpectrAA-10Plus) at 324.7 nm. The calibration curves were prepared with standard solutions of Cu in the range of 10–100 mg L^−1^ and MB and 2,4-D in the range of 1–5 mg L^−1^.

Batch sorption data were used to calculate the equilibrium contaminant uptake using Eq. (1) and removal efficiency using Eq. (2)

(1) $$q_{\exp}=\frac{C_{i}-C_{f}}{m}\times V$$

(2) $$Removal\;efficiency\;\left(\%\right)=\frac{C_{i}-C_{f}}{C_{i}}\times 100$$

where *q*_exp_ (mg g^−1^) is the equilibrium amount adsorbed on the adsorbent, *V* (L) is the sample volume, *C*_i_ (mg L^−1^) is the initial pollutant concentration, *C*_f_ (mg L^−1^) is the equilibrium pollutant concentration, and *m* (g) is the weight of the activated carbon.

### 2.5 Modeling studies

#### 2.5.1 Sorption kinetic modeling studies

The experimental adsorption capacity data (qexp, mg g^−1^) over time (t, min^−1^) were fitted using Elovich, pseudo-first-order (PFO), and pseudo-second-order (PSO) rate equations to characterize the sorption kinetics, as presented in Table 1.

**Table 1:** Correlated kinetic model constants.

Eq.	Isotherm model	Model equation	Model constant	Units
3	Elovich	$q_{t}=\frac{1}{a}\ln\;\;\ln\;\left(1+abt\right)$	**a**	mg g^−1^ min^−1^
			**b**	g mg^−1^
4	PFO	$q_{t}=q_{1}\left(1-\exp\left({-k}_{1}t\right)\right)$	**q**_**1**_	mg g^−1^
			**k**_**1**_	min^−1^
5	PSO	$q_{t}=\frac{t}{\left(\frac{1}{k_{2}q_{2}^{2}}\right)+\left(\frac{t}{q_{2}}\right)}$	**q**_**2**_	mg g^−1^
			**k**_**2**_	g mg^−1^ min^−1^

#### 2.5.2 Sorption equilibrium isotherm modeling studies

The experimental equilibrium data underwent analysis using two-parameter (Langmuir and Freundlich) and three-parameter sorption isotherm models (Redlich–Peterson, Toth, and Sips). The parameters for these isotherm model equations were identified using the MATLAB R2023a Curve Fitting Tool, and the results are presented in Table 2.

**Table 2:** Fitted isotherm model constants.

Eq.	Isotherm model	Model equation	Model constant	Units
6	Langmuir	$q_{e}=\frac{q_{m}K_{a}C_{f}}{1+K_{a}C_{f}}$	**q**_**m**_	mg g^−1^
			**K**_**a**_	L mg^−1^
7	Freundlich	$q_{e}=K_{F}{C_{f}}^{1/n}$	**K**_**F**_	(mg/g)(L/mg)^1/n^
			**N**	
8	Redlich-Peterson	$q_{e}=\frac{K_{RP}C_{f}}{1+a_{RP}{C_{f}}^{\beta }}$	**K**_**RP**_	L mg^−1^
			**a**_**RP**_	L^β^/mg^β^
			**β**	**-**
9	Toth	$q_{e}=\frac{q_{m}b_{T}C_{f}}{{\left[1+{\left(b_{T}C_{f}\right)}^{1/n_{T}}\right]}^{n_{T}}}$	**q**_**m**_	mg g^−1^
			**b**_**T**_	L g^−1^
			**n**_**T**_	**-**
10	Sips	$q_{e}=\frac{q_{m}K_{eq}{C_{f}}^{n}}{1+K_{eq}{C_{f}}^{n}}$	**q**_**m**_	mg g^−1^
			**K**_**eq**_	L g^−1^
			**n**	**-**

In these equations, *q*_*m*_ represents the maximum adsorption capacity (mg g^−1^), K_a_ is Langmuir constant (L mg^−1^), *K*_*F*_ is Freundlich constant ((mg/g)(L/mg)^1/n^), 1/*n* heterogeneity factor (dimensionless), $K_{RP}$ and $a_{RP}$ are Redlich-Peterson constants (L mg^−1^, L^β^/mg^β^), β is Redlich-Peterson exponent (between 0 and 1), *q*_*T*_ is Toth maximum adsorption capacity (mg g^−1^), *b*_*T*_ is Toth constant (L mg^−1^), *n*_*T*_ is Toth exponent (dimensionless), $K_{eq}$ is Sips constant (L g^−1^), *q*_*e*_ is the equilibrium adsorption capacity (mg g^−1^) and *C*_*f*_ is the equilibrium concentration (mg L^−1^).

The fitting accuracy between the experimental data and theoretical models was assessed using the correlation coefficient R^2^, the adjusted R^2^, the root mean square error (RMSE), and the sum of squared errors (SSE). RMSE and SSE are defined as by Eqs. (11), (12):

(11) $$RMSE=\sqrt{\frac{1}{N}\sum_{i=1}^{N}{\left(q_{cal}-q_{\exp}\right)}^{2}}$$

(12) $$SSE=\frac{1}{N}\sum_{i=1}^{N}{\left(q_{\exp}-q_{cal}\right)}^{2}$$

Here, the subscripts “exp” and “cal” denote experimental and calculated values, respectively, with N representing the number of experimental data points. A more favorable fit is indicated by lower SSE and RMSE values, coupled with a higher correlation coefficient R^2^.

#### 2.5.3 Thermodynamic modeling studies

The standard change in Gibbs free energy (ΔG°, kJ mol^−1^) can be computed from Eq. (13). The relationship between Gibbs free energy and the standard change in enthalpy (ΔH°, kJ mol^−1^) and the standard change in entropy (ΔS°, kJ mol^−1^ K^−1^) is described in Eq. (14). From the combination of Eqs. (13), (14), a linear expression is derived (Eq. (15)). From this linear form, ΔH° and ΔS° can be determined from the slope and intercept by plotting ΔG° vs 1/T graph.

(13) $$\Delta G{}^{\circ}=‐RTln\left(K_{0}\right)$$

(14) ΔG°=ΔH°‐TΔS°

(15) $$\ln\left(K_{0}\right)=\frac{\Delta S{}^{\circ}}{R}-\frac{\Delta H{}^{\circ}}{RT}$$

where $K_{0}$ is the adsorption equilibrium constant (dimensionless), T is the temperature (K), and R is the universal gas coefficient (8.31 × 10^−3^ kJ mol^−1^ K^−1^).

### 2.6 Experimental design and statistical analysis

Regarding the experimental design, the impact of two variable factors—temperature ranging from 30 to 50 °C (X_1_) and initial pollutant concentration ranging from 10 to 300 mg L^−1^ (X_2_)—on adsorption capacity was investigated using the face-centered (α = 1) Central Composite Design (CCD) of the Response Surface Methodology (RSM). This investigation was carried out using Design Expert® 12.0.1.0 software (Stat-Ease, Inc., Minnesota, USA, 2019). All experiments were concurrently conducted, and no blocking was implemented. Additional runs were included alongside those specified in the experimental design to explore a broader distribution of data.

Out of all the outcomes, only the adsorption capacity was correlated with the independent operating variables and expressed in terms of a second-order polynomial equation (Eq. (17)):

(17) $$Y=a_{0}+\sum a_{i}X_{i}+\sum a_{ii}{X_{i}}^{2}+\sum a_{ij}X_{i}X_{j}$$

In this context, *a*_*0*_*, a*_*i*_*, a*_*ii*_, and *a*_*ij*_ denote constant, linear, quadratic, and interaction coefficients, while *X*_*i*_*, X*_*i*_^*2*^, and *X*_*j*_ represent the linear, quadratic, and interaction effects of factors, respectively. The model's significance and appropriateness were assessed at a 95 % confidence interval through the analysis of variance (ANOVA).

## 3 Results and discussion

### 3.1 Structural characterization of the ACs

#### 3.1.1 Elemental (CHNS) analysis

The elemental analysis results of the produced and commercial ACs are given in Table 3. The typical mass composition of an olive stone as a raw material consists of carbon (≈43–50 wt%), oxygen (≈43–49 wt%), hydrogen (≈6–7 wt.%) and low nitrogen and sulfur content (≤0.4–0.1 wt%) [12]. The high carbon rates obtained in all the AC samples are an indication that activated carbon has been successfully obtained from the raw olive stones. The samples with the highest C content were determined as PAC (88.25 %) and OSAC (82.36 %) followed by ZOSAC (79.10 %), HOSAC (75.01 %), KOSAC (71.32 %), and GAC (65.31 %). No significant differences were observed in H, N and S contents. The percentages of sulfur and nitrogen were relatively low, but they were inevitably released as NO_x_ and SO_x_ gases as a result of combustion. The low ash percentages in Table 3 indicated that as a result of combustion, the biomass did not remain as ash, but was almost completely converted into activated carbon in all modifications.

**Table 3:** Elemental compositions of all ACs.

Activated carbon	C (%)	H (%)	N (%)	S (%)	Ash content (%)
OSAC	82.36	2.04	0.74	0.42	3.70
HOSAC	75.01	2.27	0.48	0.40	6.65
KOSAC	71.32	2.30	0.45	0.54	3.49
ZOSAC	79.10	1.77	0.83	0.24	6.40
PAC	88.25	1.23	0.35	0.36	4.00
GAC	65.31	0.36	0.64	0.34	7.00

#### 3.1.2 SEM analysis

**Fig. 1:**
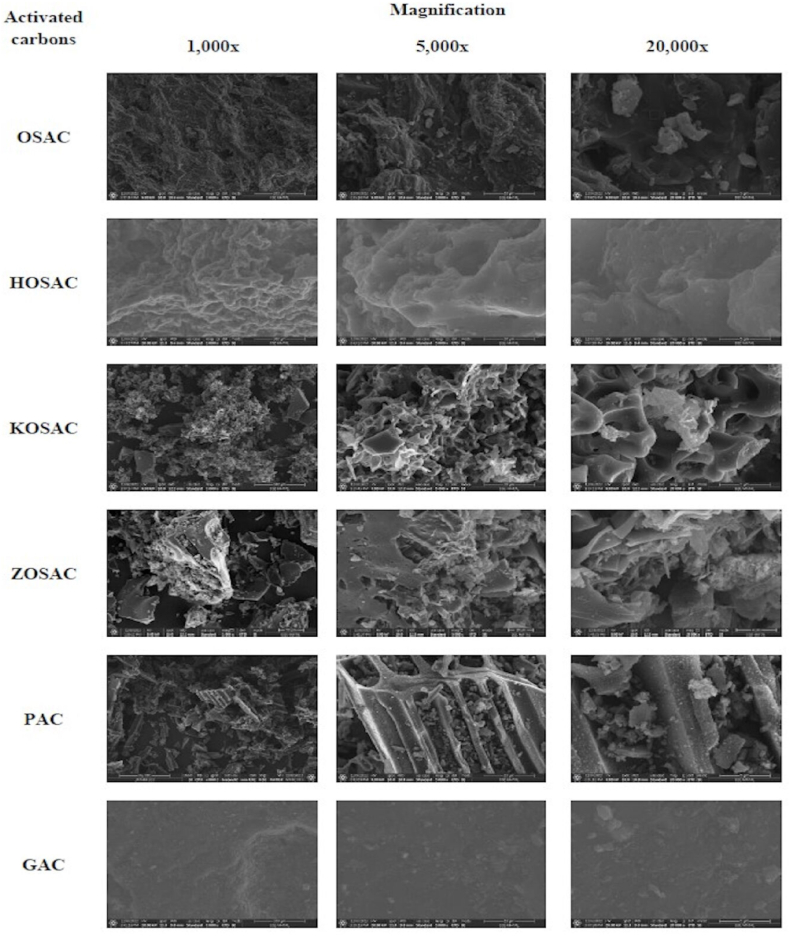
SEM micrographs of all the ACs.

The surface morphologies of all activated carbons were examined using SEM analysis. SEM images at 1,000x, 5,000x and 20,000x magnification are given in Fig. 1. The OSAC presented a smooth surface lacking a developed porous structure, with only some micropores and occasional cracks and curves resulting from pyrolysis without chemical treatment. After activation, the surfaces of the OSAC transformed, losing their smoothness and revealing irregularities and heterogeneous pore structures. This suggests that, during impregnation, molecules from the chemical activator in HOSAC, KOSAC, and ZOSAC permeated the texture of the olive stone. Subsequently, the activating agent evaporated, leaving behind a porous carbon structure [13]. A comparison of the precursor and HOSAC morphologies highlighted significant changes induced by activation. The HOSAC sorbent surface exhibited a prominently microporous structure, indicating the highly developed surface area of this material. Acid activation in certain regions converted micropores into mesopores (Fig. 1). In contrast, the acid-activated carbon displayed a greater number of mesopores, while the KOSAC predominantly featured a microporous structure with a high surface area. The external surface of the ZOSAC was entirely covered with cavities, showcasing the presence of mesopores within each oval section. In the PAC sample, a tubular structure was observed, distinct from all other activated carbons, which featured both micropores and mesopores. On the other hand, GAC displayed a rough surface with oval patterns similar to the OSAC.

#### 3.1.3 BET analysis

**Fig. 2:**
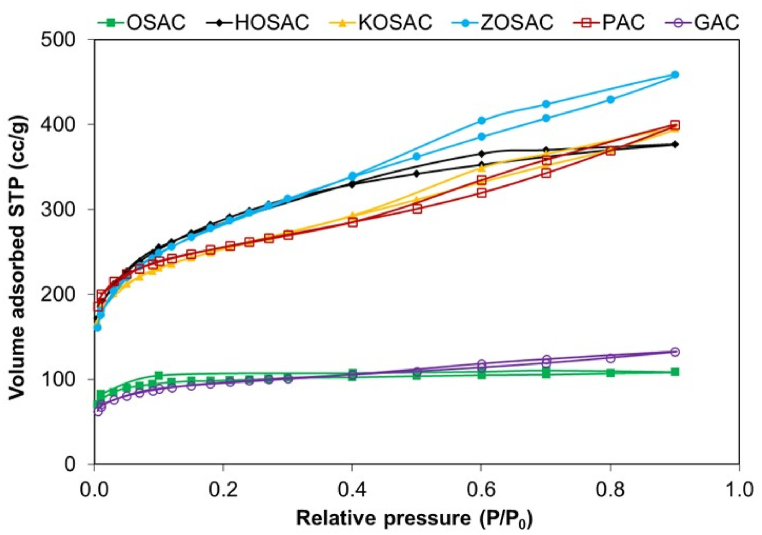
BET isotherm linear plots of all the ACs.

The surface areas and pore size distributions of the activated carbons were investigated using BET analysis. The BET adsorption-desorption isotherms are given in Fig. 2. When the isotherms are examined, it is possible to identify the type of pores present in the adsorbents by evaluating the type of isotherm curves according to IUPAC classification. OSAC and GAC resembled the Type I isotherm since the Type I isotherm follows a horizontal path representing a material with a microporous structure. It showed the formation of highly porous materials with predominantly narrow pore size distribution occurring at low relative pressures. There is no capillary condensate along the path and therefore no hysteresis loops were formed [14]. This type of isotherm characteristically belongs to adsorbents with a large number of small pores like in OSAC and GAC. On the other hand, the isotherms obtained for HOSAC, KOSAC, ZOSAC and PAC were similar to the Type IV isotherm, suggesting the prevalence of mesoporous particle size distribution. The existence of the H3 hysteresis loop type is indicative of slit-shaped pores as defined in the study of Raupp et al. [15].

**Fig. 3:**
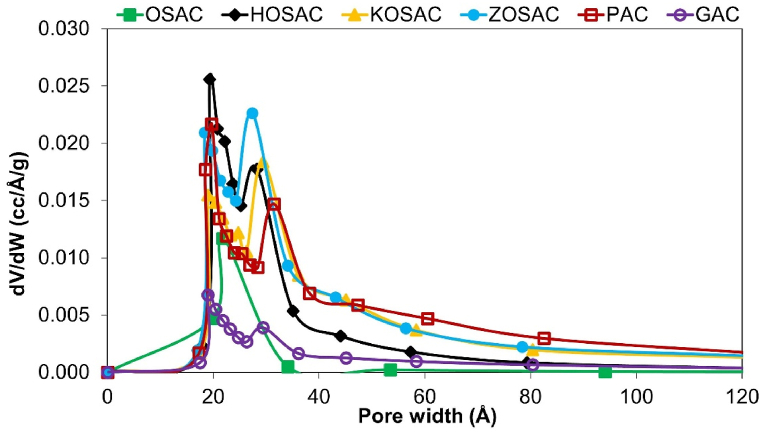
Pore size distributions of all the ACs.

The pore size distribution of all the AC samples was evaluated according to the standard ASTM 4641 using the Barrett–Joyner–Halenda (BJH) method shown in Fig. 3. When the pore size distribution graph of all AC samples is examined, it is seen that the pore distribution of the OSAC and GAC is nearly 20 Å, whereas in other samples, it is between 20 and 50 Å. Pores in OSAC and GAC typically have a microporous structure that, once filled with adsorbate, leaves little or no external surface for further adsorption. For HOSAC, KOSAC, ZOSAC and PAC, in addition to having developed micropores, they also have a predominantly mesoporous structure.

The BET, Langmuir and t-plot external surface areas, t-plot micropore area, t-plot micropore, and the BJH adsorption cumulative pore volumes (calculated by measuring adsorbed N_2_ amounts at a relative pressure of 0.95) of all the ACs are given in Table 4. The determination of mesopore volumes was achieved by subtracting the micropore volumes from the total pore volumes. A well-developed porous network comprising all pore sizes improves the adsorption properties of activated carbon. In general, the contribution of the macropore system to the total surface area and adsorption capacity of the activated carbon is very minimal because excess pore volume reduces density loss. On the other hand, high macropore and mesopore percentages may be useful since they enable rapid adsorbate transport toward the interior of the material before subsequent diffusion into the micropores during adsorption.

**Table 4:** Surface areas, pore areas and volumes of produced and activated carbons.

Activated carbon	BET surface area (m^2^ g^−1^)	t-plot micropore area (m^2^ g^−1^)	t-plot external surface area (m^2^ g^−1^)	Langmuir surface area (m^2^ g^−1^)	t-plot micropore volume (cm^3^ g^−1^)	BJH adsorption cumulative pore volume (cm^3^ g^−1^)
OSAC	341.047	203.285	138.077	454.214	0.094	0.148
HOSAC	970.193	242.564	727.628	1479.187	0.125	0.359
KOSAC	837.790	331.877	505.913	1261.575	0.171	0.461
ZOSAC	973.078	189.779	783.299	1492.403	0.096	0.512
PAC	824.246	441.958	382.287	1228.213	0.228	0.511
GAC	311.173	149.593	161.580	466.016	0.077	0.131

The BET surface areas in decreasing order are as follows: S_BET, ZOSAC_ (973.078 m^2^ g^−1^**)** > S_BET, HOSAC_ (970.193 m^2^ g^−1^) > S_BET, KOSAC_ (837.790 m^2^ g^−1^) > S_BET,PAC_ (824.246 m^2^ g^−1^) > S_BET, OSAC_ (341.047 m^2^ g^−1^) > S_BET,GAC_ (311.173 m^2^ g^−1^). Parallel results were obtained for the t-plot external surface area and Langmuir surface area which is indicates monolayer capacity and capillary condensation in mesopores. First of all, it was observed that all the impregnated AC samples have larger surface areas compared to the commercial and raw precursor which shows the success of the chemical activation and carbonization. Secondly, the raw olive stones seemed to have a unique porosity that provides the necessary nutrients for the plant from the body to the leaves, flowers, and stones above by channels with these types of pores. But still, it has a higher surface area compared to the commercial GAC and some other literature studies such as the study done by Korkmaz and Onal [16] with raw hemp with a BET surface area of 171.75 m^2^ g^−1^.

Typically, higher surface area results in higher adsorption capacity probably due to the opening of the restricted pores; therefore, the direct use of agricultural waste without any activation has lower adsorption efficiency since it does not provide the necessary pore structure, which is the case with OSAC. The surface area results obtained are very much compatible with the ones in the literature. The BET surface area of HOSAC (970.193 m^2^ g^−1^) is superior when compared with the study done by Pehlivan [17] with the pomegranate pulp activated by H_3_PO_4_ having 2/1 impregnation ratio and 700 °C temperature with a surface area of 869 m^2^ g^−1^. Also, it is seen that the ZOSAC has a larger surface area (973.078 m^2^ g^−1^) when compared to the study of Raupp et al. [15] The process involved the utilization of chemically activated olive pomace in a ratio of 1:0.8:0.2 for olive pomace, zinc chloride, and calcium hydroxide, followed by thermal activation through pyrolysis at 550 °C for 30 min. Furthermore, in a study conducted by Diaz et al. [12] involving olive stones, activation with H3PO4 led to the formation of a predominantly microporous material with a notably higher presence of mesoporosity, resulting in achieving BET surface area values of 1100 m^2^g^-1^. Activation with KOH led to the development of high porosity, primarily microporous, with BET surface area values reaching around 2000 m^2^g^-1^.

Apart from the surface area, the area of the micropores and mesopores is also especially important. In particular, the presence of meso- and micropores in activated carbon improves the adsorption of larger molecules. The more mesopores with an average pore diameter of 2–10 nm increase, the more the surface area of the activated carbon will increase. In Tables 4 and it seems that PAC and KOSAC have the highest t-plot micropore area and volume which shows better adsorption potential for smaller molecules such as Cu, whereas ZOSAC has the least micropore volume among all the impregnated AC which signals better adsorption performance for larger molecules such as MB and 2,4-D with higher mesoporosity. The porosity of the materials may decrease due to the blockage of the internal porosity by incorporated functional groups. Moreover, after chemical activation, micropores might have been enlarged and converted into meso and macropores leading to a decrease in specific surface area.

#### 3.1.4 XRD analysis

**Fig. 4:**
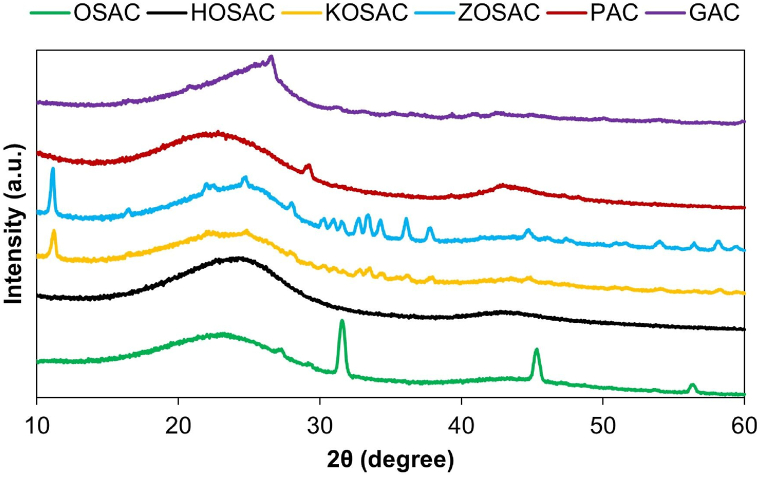
XRD graphs of all the ACs.

The microstructure characterization of the activated carbons was conducted using XRD analysis and is given in Fig. 4. Generally, in all of the samples, broad diffraction peaks located between 2θ 20°–30° and 40°–50° confirmed that the structure is more likely amorphous. The only small peak present (2θ, 29.3 °C) in the XRD pattern of commercial PAC belonged to the carbon disulfide proved by the specification sheet of Merck indicating that the total sulfur amount is ≤ 5000 mg/kg (JCPDS 01-076-0063). The other commercial AC which is in granular form (GAC) has a % intensity of 100 % at an angle of [°2θ] 26.60 matching with carbon (JCPDS 00-025-0284, 26.61 matching with graphite (JCPDS 01-075-2078) and 33.0 matching with carbon tetrachloride (JCPDS 00-050-1911). The XRD spectrum of untreated OSAC reveals a predominantly amorphous structure, characterized by a broad peak with a peak maximum at 23° = 2θ, as determined by Zakir Hossain et al. [18] in a study involving hemp bark. Similarly, it can be inferred that cellulosic units are integrated into the structure as distinct macromolecular groups, given that olive stone is well-established as a lignocellulosic material, composed primarily of hemicellulose, cellulose, and lignin. The % intensity of OSAC is 100 % at an angle of [°2θ] 29.3, corresponding to carbon disulfide (JCPDS 01-076-0063) inherent to the olive stone structure. In addition to the biomass-based carbon peaks, residual peaks persist, associated with chemical agents like NaCl. These remnants may be attributed to incomplete removal during the washing processes intended to neutralize the produced activated carbon, as noted in the study by Rahma et al. [19] The 2θ values corresponding to the NaCl are 27.4, 31.7, 45.4 and 56.5, respectively (JCPDS 01-077-2064). The presence of a broad diffraction background and the absence of a distinct peak in the HOSAC sample indicated a primarily amorphous structure. The XRD pattern of HOSAC displayed a significant peak of amorphicity around 26°, resembling the hump characteristic of exfoliated graphite crystals, as evidenced by the peaks at 2θ, 23.7. This can be interpreted as raw olive stone structurally transforming into graphite/graphene structure as a result of H_3_PO_4_ activation (JCPDS 01-075-2078; [18]).

In the XRD traces of KOH, it is seen that the peaks become narrower and wider. Besides the main amorphous structure of the activated carbon, the characteristic peaks belonging to the crystal structures of zinc hydroxide chloride hydrate (Zn_5_(OH)_8_Cl_2_•H_2_O) Rhombohedral phase. The 2θ values corresponding to the structure are 11.2 with a % intensity of 100 %, 16.6, 22.1, 24.8, 28.1, 30.4, 32.8, 33.5, 34.4, 36.0, 36.3, 37.9, 43.8, 44.8, 46.0, 50.9, 51.7, 53.9, 57.0, 58.2 and 59.4, respectively (JCPDS 01-072-1444).

The XRD pattern of ZOSAC is similar to the KOSAC sample. The 2θ values corresponding to the Zn_5_(OH)_8_Cl_2_•H_2_O are 11.2 with a % intensity of 100 %, 16.6, 22.1, 22.5, 24.8, 28.1, 30.4, 31.1, 32.8, 33.5, 34.4, 37.9, 43.7, 44.8, 46.0, 53.9, 58.2 and 59.4, respectively (JCPDS 01-072-1444; [[20], [21], [22]]). Additionally, ZnO has a main peak at 2θ, 36.2 °C. The 2θ values for this structure are 31.7, 34.4, 36.2, 47.5 and 56.5, respectively (JCPDS 01-079-00207; [23]).

#### 3.1.5 pH_PZC_ analysis

**Fig. 5:**
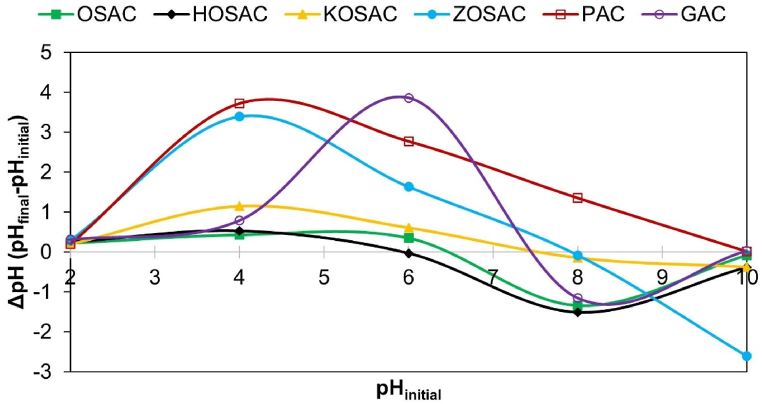
pH_PZC_ results of all the ACs.

The sorption capacity is influenced by the surface charge of the adsorbent. pHPZC analysis allows for the assessment of the adsorbent's surface charge concerning pH, indicating the point at which the surface charge reaches zero and surface functional groups cease to affect the solution's pH [15]. Fig. 5 illustrates the surface charges of all activated carbons as a function of pH. When the pH of the solution in contact with the respective adsorbent was below the pHPZC values, the activated carbons exhibited a positive charge due to the protonation of the functional groups. This condition favored the adsorption of anionic species such as the 2,4-D herbicide at pH > 2.64 [24]. On the other hand, when the pH of the solution is higher, it means that the adsorbent is negatively charged, which favors cationic species adsorption [25]. The pH_PZC_ values and zeta potentials (mV) of all the ACs are listed in Table 5. The pH_PZC_ values of OSAC, HOSAC, KOSAC, ZOSAC, PAC and GAC were found as 6.5, 6.0, 7.5, 8.0, 10.0 and 7.4, respectively which demonstrate that OSAC and HOSAC have an acidic surface nature since a pH_pzc_ < 7 signals the dominant of acidic groups over basic groups, whereas the vice versa is valid for other ACs having a basic surface nature. This indicated that OSAC and HOSAC are more efficient adsorbents for the removal of cationic dyes such as MB and cationic heavy metals such as Cu. At low pH levels, the adsorption process is hindered by the electrostatic repulsion between the positively charged surface of the activated carbon and both the cationic MB dye and Cu molecules. Furthermore, an excess of hydrogen ions in this environment may compete with MB and Cu molecules for the available adsorption sites. With an increase in pH, deprotonation of the activated carbon surface occurs, leading to a negative charge and facilitating the adsorption of MB. Consequently, the adsorption capacity for MB increases, and it is expected that this trend will persist when the solution pH surpasses 6.5. However, this pattern does not hold true for Cu adsorption, as the amount of Cu adsorbed increases when the pH decreases to 5. This discrepancy could be attributed to other factors, such as the pore size of the adsorbent playing a crucial role in Cu removal, alongside the mechanism of electrostatic interactions, as explained in the study by Patawat et al. [26] carried out using activated carbons prepared from the *Dipterocarpus alatus* fruit, by chemical activation using ZnCl_2_, H_3_PO_4_ and KOH for MB removal. These results also correspond to the study of Egirani et al. [27] with PAC and GAC with a pH_PZC_ value of 7.01 obtained from the shells of a palmae biomass and used in mercury ion removal.

**Table 5:** pH_PZC_ and zeta potential measurement values of all the ACs.

Activated carbon	pH_PZC_	Zeta Potential (mV)		
		pH = 3	pH = 5	pH = 8
OSAC	6.5	−8.01	−27.74	−31.04
HOSAC	6.0	−5.75	−15.06	−19.85
KOSAC	7.5	−12.89	−15.84	−16.00
ZOSAC	8.0	−4.45	−5.40	−5.75
PAC	10.0	−11.27	−17.38	−18.90
GAC	7.4	−15.22	−13.47	−13.50

### 3.2 Adsorption studies

#### 3.2.1 Effect of the contact time on adsorption performance

**Fig. 6:**
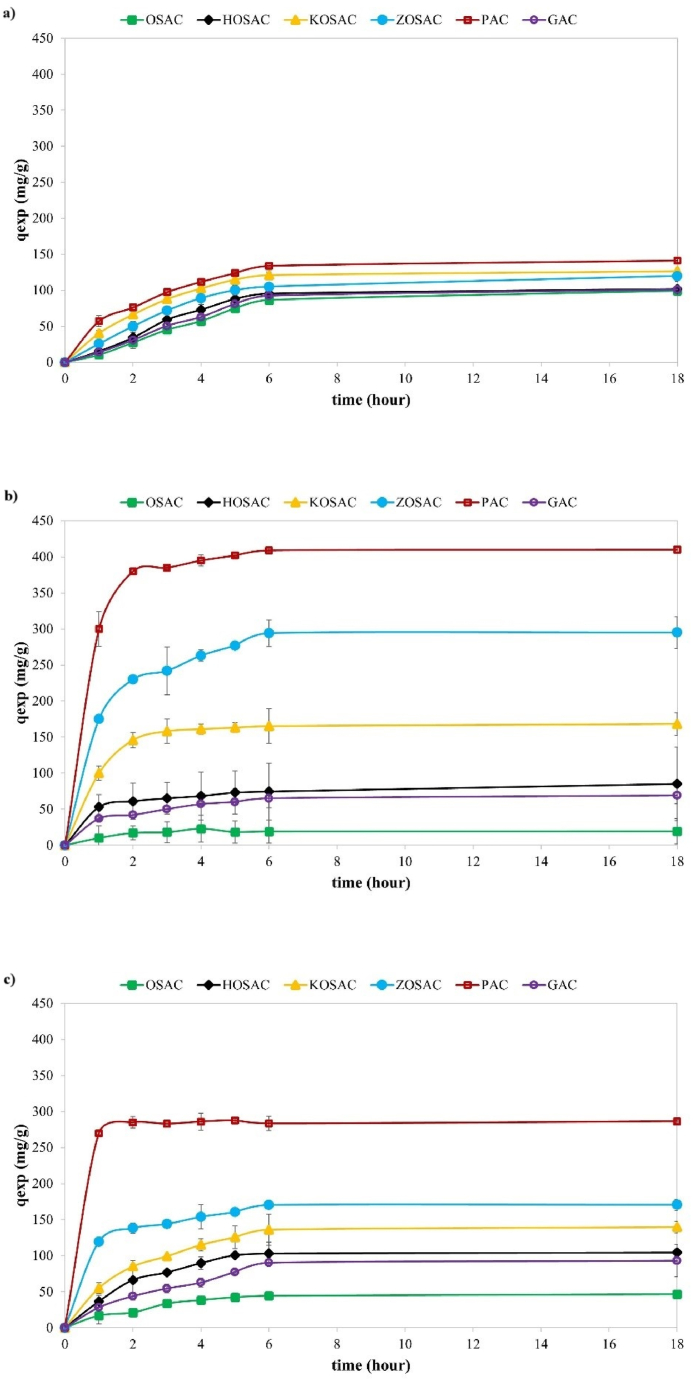
The effect of contact time on **a)** Cu, **b)** MB, and **c)** 2,4-D adsorption.

The effect of contact time on adsorption performance was studied in the range of 1–18 h for Cu, MB and 2,4-D adsorption as shown in Fig. 6. Similar interactions were seen with the three different types of contaminants. The initial phase corresponds to robust and rapid adsorption, facilitated by the abundance of vacant sites on the material surface, simplifying the uptake of contaminants with minimal competition for these sites. Additionally, during the early stages of adsorption, active sites on the surface of activated carbons are more accessible, and the concentration gradient is higher [28,29]. While the adsorption capacity gradually increased from 1 to 6 h, beyond the 6-h mark, there was no substantial difference in adsorption capacity. A slower phase ensued, signifying a gradual reduction in the availability of free adsorption sites on the activated carbon surfaces.

The Cu adsorption capacities of the ACs can be listed from the highest to lowest as follows: q_PAC_ (145.40 mg g^−1^) > q_KOSAC_ (125.20 mg g^−1^) > q_ZOSAC_ > q_HOSAC_ > q_GAC_ > q_OSAC_. The MB adsorption capacities of the ACs can be listed from the highest to lowest as follows: q_PAC_ (409.14 mg g^−1^) > q_ZOSAC_ (294.35 mg g^−1^) > q_KOSAC_ > q_HOSAC_ > q_GAC_ > q_OSAC._ The 2,4-D adsorption capacities of the ACs can be listed from the highest to lowest as follows: q_PAC_ (283.40 mg g^−1^) > q_ZOSAC_ (170.33 mg g^−1^) > q_KOSAC_ > q_HOSAC_ > q_GAC_ > q_OSAC_. As anticipated, given the variation in specific surface areas, the amount of adsorbed heavy metal ions and herbicide-dye molecules on the PAC was higher than on all the other materials. The other activated carbons displayed comparable trends to MB and 2,4-D, with minor distinctions that could be ascribed to diverse functional groups on the carbon surface or variations in the accessibility of the adsorption surface to Cu^2+^ ions. For subsequent adsorption studies, the equilibrium time was determined to be 6 h. Considering their notable adsorption capacities, KOSAC and PAC activated carbons will be employed for Cu, ZOSAC and PAC for MB, and ZOSAC and PAC activated carbons for 2,4-D in further adsorption studies.

#### 3.2.2 Effect of contaminant concentration on adsorption performance

**Fig. 7:**
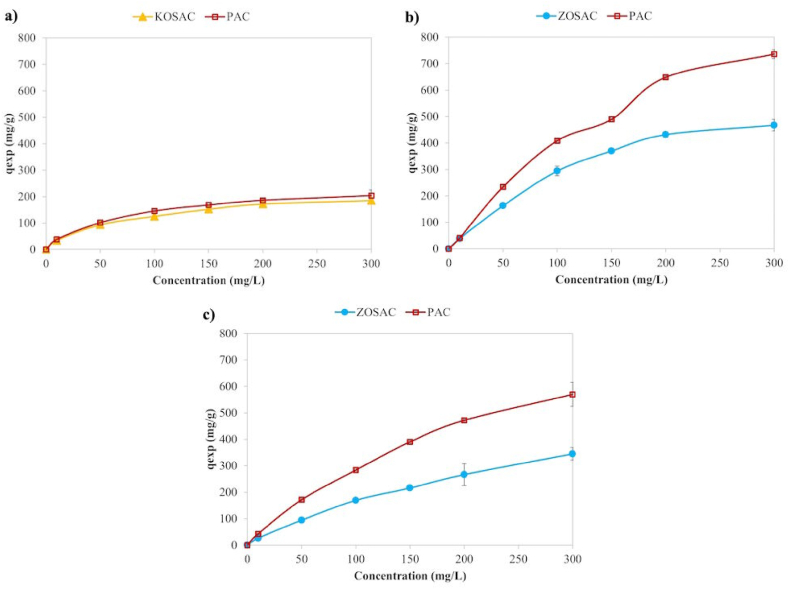
The effect of contamination concentration on **a)** Cu, **b)** MB, and **c)** 2,4-D adsorption.

The initial pollutant concentration plays an important role towards the adsorption capacity. In order to examine the effect of concentration, an isotherm study was carried out in the contaminant concentration range of 10–300 mg L^−1^. The results are given in Fig. 7 for Cu, MB and 2,4-D.

The adsorption capacity was observed to increase directly with the contaminant concentration. This can be attributed to the high contaminant concentration, which serves as a robust driving force, accelerating the mass transfer of adsorbate from the solution to the adsorbent surface. The abundant availability of adsorbate enhances the occupation of numerous vacant adsorption sites on the adsorbent's surface. In contrast, at low concentrations, there were limited adsorbate ions available to occupy the interface of the activated carbons. The contaminant uptake approached a saturation plateau for both activated carbons across all concentrations. This phenomenon occurred because, under the specified contact time and pH conditions, the active sites on the adsorbent's surface were completely occupied due to the adsorbent dose utilized when exposed to contaminant concentrations of 300 mg L^−1^. An alternative explanation could be that with elevated contaminant concentrations, there is an increased abundance of molecules, leading to a higher frequency of collisions between the contaminant and activated carbon (AC). Similar trends were observed in the studies of Batool and Valiyaveettil [30] for MB and neutral red, Hou et al. [31] for Rhodamine B, Tang et al. [32] for MB, Fita et al. [33] for Cu^2+^ and Mohd Noor Hazrin et al. [34] for 2,4-D adsorption. Below the equilibrium concentration, the lower zeta potential like in the case of OSAC caused electrostatic repulsion between the AC and the 2,4-D anions under neutral conditions as tabulated in Table 5. As the zeta potential became negative, the removal rate of 2,4-D by activated carbon decreased with the reduction of the zeta potential. Beyond the equilibrium concentration, the specific surface area played a crucial role in increasing the adsorption capacity [24].

#### 3.2.3 Effect of temperature on adsorption performance and thermodynamic constants

**Fig. 8:**
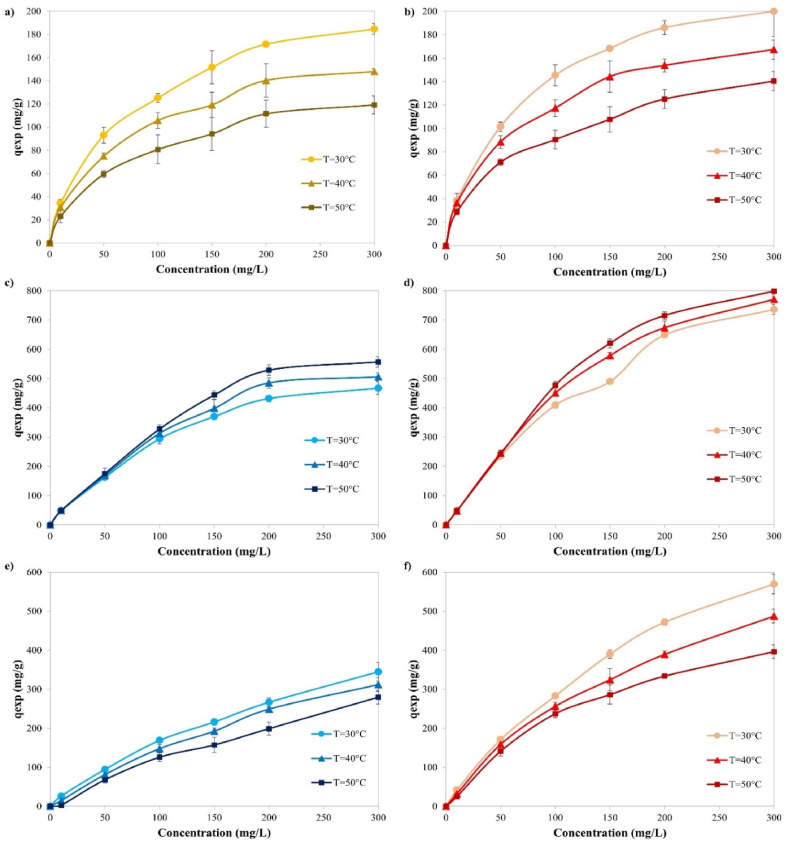
The effect of temperature on adsorption for **a)** KOSAC-Cu, **b)** PAC-Cu, **c)** ZOSAC-MB, **d)** PAC-MB, **e)** ZOSAC-2,4-D, and **f)** PAC-2,4-D.

The effect of temperature on adsorption capacity was investigated at three different temperature levels (30, 40, and 50 °C). The results are given in Fig. 8 for Cu, MB and 2,4-D, respectively. While the temperature increase affected the adsorption of Cu and 2,4-D inversely, it influenced the adsorption capacity directly for MB. Moreover, it was observed that the adsorbed amount was favored by decreasing the temperature, yielding the highest capacities at 30 °C and indicating exothermic adsorption behavior for Cu and 2,4-D. The impact of temperature can be explained in terms of solubility, as there is a tendency for copper ions to shift from the solid phase to the bulk phase with an increase in solution temperature. As the temperature rises, the solubility of 2,4-D and Cu increases, making them more soluble and less prone to migrate to the adsorbent surface and be adsorbed, resulting in reduced adsorption at elevated temperatures. These results are compatible with the studies conducted by Tang et al. [32] using wastepaper-based activated carbon for MB adsorption and Georgin et al. [28] using sulfuric acid-treated *Physalis peruviana* fruit for 2,4-D adsorption.

To investigate the thermodynamic behavior of adsorption, Gibbs free energy change *(*ΔG°*)*, standard change in enthalpy *(*ΔH°*)* and the standard change in entropy *(*ΔS°*)* constants were calculated. The results obtained for Cu, MB and 2,4-D adsorption are given in Table 6. The negative ΔG° values for all cases of adsorption demonstrate the feasibility and spontaneity of the adsorption. The positive values of ΔS° for MB adsorption is related to the increase in randomness at the activated carbon-solution interface during the dye adsorption, whereas negative entropy values for Cu and 2,4-D adsorption reveal that randomness decreases due to the relationship between the adsorbate and adsorbent.

**Table 6:** Thermodynamic constants for Cu, MB and 2,4-D adsorption.

Cu adsorption					
Adsorbent	Temperature (°C)	ΔG° (kJ/mol)	ΔH° (kJ/mol)	ΔS° (kJ/mol K)	R^2^
KOSAC	30	−2.77	−19.28	−0.0541	0.945
	40	−2.46			
	50	−1.69			
PAC	30	−3.14	−15.25	−0.0397	0.839
	40	−3.04			
	50	−2.34			
**MB adsorption**					
ZOSAC	30	−2.08	1.76	0.0213	0.8673
	40	−2.82			
	50	−2.95			
PAC	30	−4.65	10.90	0.0432	0.8564
	40	−5.00			
	50	−5.07			
**2,4-D adsorption**					
ZOSAC	30	−1.24	−31.00	−0.0982	0.999
	40	−0.24			
	50	0.72			
PAC	30	−2.98	−19.94	−0.0561	0.994
	40	−2.34			
	50	−1.85			

As per earlier studies, adsorption can be regarded as physisorption when *ΔH°* is less than 20 kJ/mol, and it can be considered as chemisorption when *ΔH°* falls within the range of 80–200 kJ/mol [35]. Based on this assumption, since the ΔH° values of ZOSAC-2,4-D adsorption was higher than 20 kJ/mol, the adsorption can be described as chemisorption and exothermic. Also, the findings indicate that the adsorption mechanism responsible for copper removal is primarily physical. The adsorption process occurs through electrostatic interactions, typically associated with low adsorption heat suggesting that the adsorption process is inherently exothermic in nature as discussed by Anitha et al. [36]. On the other hand, the positive value of ΔH° for MB adsorption shows the nature of the adsorption is endothermic.

#### 3.2.4 Effect of pH on adsorption performance

**Fig. 9:**
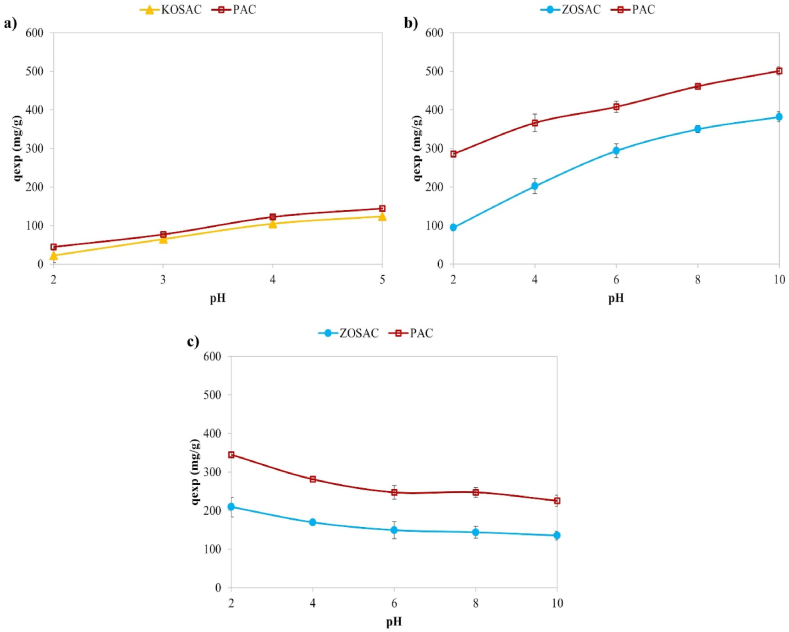
The effect of pH on **a)** Cu, **b)** MB, and **c)** 2,4-D adsorption.

Another parameter that affects adsorption capacity is pH. In order to see the effect of pH on the adsorption capacity, pH ranges of 2–5 for Cu and 2–10 for MB and 2,4-D were studied. Since the NaOH solution added to increase the pH of the copper solution reacted with the copper after a certain point and formed copper hydroxide, the maximum pH reached could be up to 5. The results are given in Fig. 9 for Cu, MB, and 2,4-D. It was observed that the adsorption capacity of Cu and MB increased as the pH increased, while it decreased for 2,4-D. The highest adsorption capacities were observed at pH 5 for Cu (KOSAC: 124.10 mg g^−1^, PAC: 144.44 mg g^−1^), pH 10 for MB (ZOSAC: 382.19 mg g^−1^, PAC: 500.79 mg g^−1^), and a pH 2 for 2,4-D (ZOSAC: 209.46 mg g^−1^, PAC: 345.12 mg g^−1^). Darweesh et al. [37] and Neisan et al. [38] also achieved higher Cu adsorption capacity at pH 5. For MB adsorption, Ramutshatsha-Makhwedzha et al. [39] and Jawad et al. [40] obtained the highest MB adsorption in an alkaline medium with a pH of around 10. Lazarotto et al. [41] and Mohd Noor Hazrin et al. [34] observed that the highest 2,4-D was adsorbed using an acidic medium with a pH of 2.

#### 3.2.5 Effect of the dosage of adsorbent on the adsorption performance

**Fig. 10:**
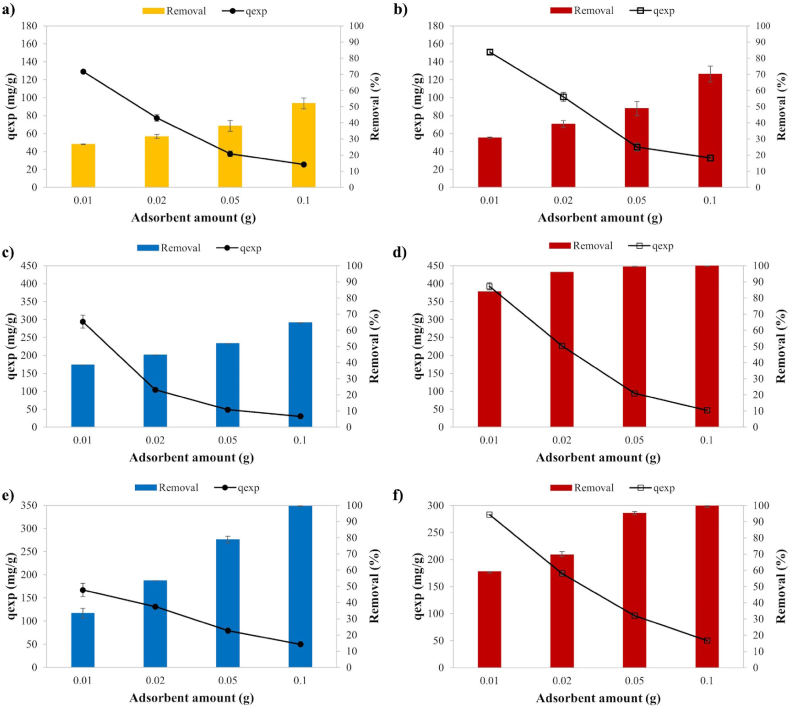
The effect of the adsorbent dosage on the adsorption for **a)** KOSAC-Cu, **b)** PAC-Cu, **c)** ZOSAC-MB, **d)** PAC-MB, **e)** ZOSAC-2,4-D, and **f)** PAC-2,4-D.

The quantity of adsorbent is a pivotal factor in the adsorption of pollutants, influencing the availability of active functional groups for adsorption. This correlation is reflected in the variations in the percentage removal of contaminants. The effect of the amount of adsorbent was investigated with different amounts of activated carbons (0.01–0.1 g) for Cu, MB and 2,4-D adsorption as shown in Fig. 10. While the Cu removal increased from 27 % to 52 % for KOSAC, the adsorption capacity decreased from 125.20 mg g^−1^ to 26.68 mg g^−1^ since it was inversely proportional to the amount of adsorbent. For PAC, the Cu removal increased from 31 % to 70 %, while the adsorption capacity decreased from 145.40 mg g^−1^ to 32.78 mg g^−1^. An escalation in the quantity of adsorbent resulted in a decline in the Cu2+ uptake capacity, as outlined in the Fita et al. [33] investigation involving hull-based activated carbon and its interaction with the copper ions. The reduction in adsorbent capacity at elevated dosages can be ascribed to a decrease in the number of available active sites per unit mass. This decrease is likely associated with the agglomeration of activated carbon particles induced by the agitation of the suspension during adsorption. As a result, this agglomeration results in a diminished spacing between adsorbent particles, consequently restricting the diffusion of Cu^2+^ ions toward the adsorption sites.

For ZOSAC, the MB removal increased from 39 % to 65 %, while the adsorption capacity decreased from 294.35 mg g^−1^ to 30.27 mg g^−1^. For PAC, the MB removal increased from 84 % to 99 %, while the adsorption capacity decreased from 409.14 mg g^−1^ to 47.17 mg g^−1^. As anticipated, a similar trend of an inverse relationship between the mass of the adsorbent and adsorption capacity was observed in Batool and Valiyaveettil's [30] study concerning MB. The effective surface area per unit mass of the absorbent diminishes as the concentration of the adsorbent increases. This decline is ascribed to the notable presence of unoccupied surface functional groups and cohesive interactions among adsorbent particles, such as aggregation or agglomeration. Furthermore, the adsorption of pollutants on the adsorbent surface is driven by electrostatic interactions and van der Waals forces between the dyes and the adsorbent surface, enhancing the efficacy of dye adsorption.

For ZOSAC, while the 2,4-D removal increased from 33 % to 99 %, the adsorption capacity decreased from 170.33 mg g^−1^ to 49.90 mg g^−1^. For PAC, the 2,4-D removal increased from 59 % to 99 %, while the adsorption capacity decreased from 283.40 mg g^−1^ to 50.32 mg g^−1^. Similarly, an increase in the adsorbent dosage increased the percentage of removal, on the other hand, a decrease in the adsorption capacity in the study of Kirbiyik et al. [42] due to the aggregation or overlapping of adsorption sites.

### 3.3 Modeling studies

#### 3.3.1 Kinetic modeling

Utilizing kinetic models is typically essential for assessing the adsorption rate of contaminants. This approach serves as a vital tool for both system design and economic considerations. Elovich, PFO and PSO kinetic models were applied to better understand the AC behavior in terms of contaminant removal. Model parameters and correlation coefficients for Cu, MB and 2,4-D adsorption are listed in Table 7. Considering the outcomes presented in Table 7, the correlation coefficient values of the Elovich and PSO kinetic models for Cu adsorption are relatively modest, ranging between 0.891 and 0.984. Furthermore, the experimental qe values do not align with the calculated values derived from the linear plots. Hence, that means the PFO model is more suitable for defining the physisorption mechanism of Cu adsorption. The k_1_ values of Cu increased in the following direction: OSAC < GAC < HOSAC < ZOSAC < KOSAC < PAC which shows exact conformity to the results obtained for the effect of contact time. In the study of Fita et al. [33], the pseudo-first-order model seemed to be more appropriate for describing the adsorption of Cu2+ on activated carbon derived from Neem biomass waste and the pseudo-second-order model for the *Hyphaene thebaica* biomass waste AC.

**Table 7:** Kinetic model constants for Cu, MB, and 2,4-D adsorption for all ACs.

Cu adsorption									
Activated carbon	Elovich			PFO			PSO		
	a (mg g^−1^ min^−1^)	b (g mg^−1^)	R^2^	k_1_ (min^−1^)	q_1_ (mg g^−1^)	R^2^	k_2_ (g mg^−1^ min^−1^)	q_2_ (mg g^−1^)	R^2^
OSAC	0.0246	0.5142	0.914	0.0034	106.187	0.959	2.18E-05	138.73	0.938
HOSAC	0.0281	0.8219	0.891	0.0045	108.625	0.961	3.24E-05	135.40	0.930
KOSAC	0.0305	2.3307	0.932	0.0064	130.267	0.995	4.96E-05	153.09	0.973
ZOSAC	0.0270	1.2043	0.935	0.0049	123.830	0.991	3.45E-05	150.72	0.969
PAC	0.0304	3.3798	0.957	0.0069	141.605	0.991	5.31E-05	163.94	0.984
GAC	0.0261	0.6386	0.905	0.0039	107.983	0.960	2.60E-04	137.77	0.935
**MB adsorption**									
OSAC	0.3547	6.5743	0.861	0.0139	19.966	0.948	9.72E-04	22.13	0.914
HOSAC	0.0882	20.483	0.998	0.0166	74.533	0.954	2.80E-04	83.86	0.988
KOSAC	0.0466	108.267	0.952	0.0160	166.247	0.998	1.40E-04	183.15	0.986
ZOSAC	0.0233	68.435	0.974	0.0139	283.929	0.985	6.63E-05	316.97	0.994
PAC	0.0288	9261.7	0.979	0.0226	402.868	0.997	1.03E-04	431.09	0.995
GAC	0.0798	4.0389	0.977	0.0095	65.693	0.966	1.80E-04	74.48	0.985
**2,4-D adsorption**									
OSAC	0.0836	0.8796	0.922	0.0064	48.069	0.984	1.35E-04	56.38	0.961
HOSAC	0.0407	2.9198	0.922	0.0076	107.669	0.994	7.63E-05	124.82	0.970
KOSAC	0.0317	3.9979	0.950	0.0075	140.519	0.995	5.94E-05	162.26	0.985
ZOSAC	0.0525	227.244	0.987	0.0193	161.319	0.977	1.81E-04	176.64	0.994
PAC	0.1103	1.4E12	0.996	0.0483	285.371	0.999	8.98E-04	289.38	0.999
GAC	0.0375	1.1332	0.935	0.0051	96.357	0.979	5.01E-05	115.02	0.963

On the other hand, and higher than those of the Elovich correlation coefficients, the results for MB and 2,4-D adsorption in Table 7 show variation between the PFO and PSO models for different activated carbons, revealing that the sorption can either be physisorption or chemisorption. In the study of Doczekalska et al. [43], it was noted that the outcomes conformed to the PSO kinetic model for 2,4-D adsorption, indicating a dependence on a diffusion-controlled system for the adsorption mechanism. The projected adsorption capacity showed an increase with the initial concentration of 2,4-D, supporting the experimental findings [28]. The k_2_ values for 2,4-D increased in the following order: KOSAC < GAC < HOSAC < OSAC < ZOSAC < PAC. The time needed to achieve equilibrium is typically associated with the volume of macro- and mesopores, which function as transporting arteries. The results are compatible with the increase in the mesopore volumes of the ACs corresponding to the faster adsorption towards the targeted herbicide. Similar results were found in the study of Batool and Valiyeveettil [30] for the adsorption of MB onto silica-coated soya waste with the adsorbent obeying the PSO kinetic model.

#### 3.3.2 Equilibrium isotherm modeling

The equilibrium study employed two-parameter models (Langmuir and Freundlich) as well as three-parameter models (Redlich-Peterson, Toth, and Sips) to investigate the adsorption mechanism. The parameters of the fitted models for the selected activated carbon are presented in Table 8, revealing that all isotherm models exhibited very high regression correlation coefficients. When two-parameter models for Cu adsorption are examined, it is seen that the Langmuir model is more suitable (KOSAC: R^2^ = 0.996, PAC: R^2^ = 0.998) as similarly found in the study of Chen et al. [44]. Similar results are valid for the MB adsorption study of Batool and Valiyaveettil [30] and for the 2,4-D adsorption study of Zhu et al. [24] which involve a higher value of correlation coefficients for the Langmuir isotherm. Remarkably, the experimental q_e_ values fell below the q_m_ values obtained from the Langmuir isotherm, indicating that within the studied concentration range, the activated carbons were not completely saturated by the pollutant molecules. The satisfactory agreement between experimental data and the Langmuir isotherm implies a consistent binding energy across the entire surface, suggesting that sorbed molecules did not engage in competition to form a monolayer. Moreover, the K_a_ values, ranging from 0.0029 to 0.0142 (0 < K_a_ < 1), affirm the favorable nature of the adsorptions [45]. Conversely, the Freundlich isotherm finds widespread application in modeling the formation of a multilayer adsorbate on a heterogeneous adsorbent surface or supporting sites with varying affinities, indicating a non-uniform energy distribution [46]. The Freundlich model was demonstrated as being the least fitting, and among all the activated carbons, the most accurate fit was achieved with ZOSAC, yielding an R^2^-adj value of 0.99809. This implies that the adsorption of 2,4-D involves multiple layers and interactions between dye molecules. Moreover, the n value ranged from 1.436 to 2.3995 (greater than 1), indicating the heterogeneous nature of the adsorbent.

**Table 8:** Isotherm model constants for Cu, MB and 2,4-D adsorption.

Isotherm model	Parameter	Cu		MB		2,4-D	
		KOSAC	PAC	ZOSAC	PAC	ZOSAC	PAC
Langmuir	**q**_**m**_**(mg g**^**−**^**^1^)**	230.2104	249.9098	727.1091	1325.40	739.0973	1101.60
	**K**_**a**_**(L mg**^**−**^**^1^)**	0.0133	0.0142	0.0066	0.0043	0.0029	0.0036
	**R**^**2**^	0.99606	0.99812	0.99518	0.99469	0.9988	0.99925
	**R**^**2**^**-adj**	0.99528	0.99774	0.99421	0.99362	0.99856	0.9991
	**RMSE**	4.8135	3.6667	14.271	22.835	4.8085	6.4747
	**SSE**	115.85	67.222	1018.3	2607.1	115.61	209.61
Freundlich	**K**_**F**_**((mg/g)(L/mg)**^**1/n**^**)**	17.2573	19.9678	20.9713	20.4855	6.5713	13.6769
	**n**	2.3506	2.3995	1.7925	1.571	1.436	1.5151
	**R**^**2**^	0.98816	0.98675	0.97491	0.98543	0.99841	0.99434
	**R**^**2**^**-adj**	0.98579	0.9841	0.96989	0.98252	0.99809	0.99319
	**RMSE**	8.3493	9.7314	32.554	37.812	5.5377	17.814
	**SSE**	348.55	473.5	5298.9	7148.9	153.33	1586.7
Redlich-Peterson	**K**_**RP**_**(L mg**^**−**^**^1^)**	4.4694	4.6072	3.5093	5.0611	2.9786	3.7803
	**a**_**RP**_**(L**^**β**^**/mg**^**β**^**)**	0.0537	0.0389	0.0001	0.0009	0.0471	0.0018
	**β**	0.8307	0.8751	1.641	1.2369	0.6173	1.1095
	**R**^**2**^	0.99812	0.99917	0.9996	0.99505	0.99954	0.9993
	**R**^**2**^**-adj**	0.99745	0.99876	0.9994	0.99257	0.99931	0.99895
	**RMSE**	3.8389	2.7201	1.6013	24.652	3.3271	6.9913
	**SSE**	58.948	29.597	84.688	2430.8	44.279	195.51
Toth	**q**_**m**_**(mg g**^**−**^**^1^)**	181.6855	215.1097	346.1055	1182.40	681.172	1040.20
	**b**_**T**_**(L g**^**−**^**^1^)**	0.0296	0.0245	0.0476	0.0035	0.0071	0.0033
	**n**_**T**_	0.8307	0.8751	0.61	1.2369	0.6173	1.1095
	**R**^**2**^	0.99833	0.99917	0.98451	0.99505	0.99954	0.9993
	**R**^**2**^**-adj**	0.99764	0.99876	0.97676	0.99257	0.99931	0.99895
	**RMSE**	3.8389	2.7201	28.6	24.652	3.3271	6.9913
	**SSE**	58.948	29.597	3271.8	2430.8	44.279	195.51
Sips	**q**_**m**_**(mg g**^**−**^**^1^)**	277.42	285.0697	582.3289	1165.00	1158.10	1102.30
	**K**_**eq**_**(L g**^**−**^**^1^)**	0.0084	0.0103	0.0102	0.0056	0.0012	0.0036
	**n**	0.7951	0.8362	1.3273	1.1022	0.8558	0.9997
	**R**^**2**^	0.99847	0.99961	0.99812	0.99504	0.99953	0.99925
	**R**^**2**^**-adj**	0.99771	0.99941	0.99718	0.99256	0.99929	0.99888
	**RMSE**	3.3546	1.8776	9.9596	24.662	3.3842	7.2389
	**SSE**	45.013	14.101	369.78	2432.9	45.811	209.61

The Redlich–Peterson model adheres to a hybrid adsorption mechanism rather than conforming to an ideal monolayer adsorption. Based on the modeling results and considering the highest R^2^ value, which surpasses 0.99 in all cases, the Redlich-Peterson model demonstrates superior performance for all activated carbons. The parameter β falls between 0 and 1 for Cu adsorption on both activated carbons and for 2,4-D adsorption on ZOSAC, while for other instances, it exceeds 1. Combining the aspects of the Langmuir and Freundlich models, the Redlich-Peterson model proves to be more comprehensive and accurate. Furthermore, this model exhibits good adaptability under both low and high concentrations of adsorbate conditions [47].

The Toth isotherm, designed as an empirical model to enhance the conventional Langmuir isotherm, is frequently advantageous in describing heterogeneous systems approaching the Henry region at infinite dilution. Conversely, the Sips isotherm model is defined by the heterogeneity factor, n. When n equals 1, it converges to the Langmuir equation, indicating a homogeneous adsorption process. Despite sharing a similar form with the Freundlich equation, the Sips model exhibits a finite limit at sufficiently high concentrations.

The optimization of an adsorption system depends on identifying the most fitting correlation for the equilibrium curves. Consistent with the Redlich-Peterson model, higher adjusted R2 and lower RMSE values were attained using the three-parameter Toth and Sips models, in contrast to the two-parameter models, for all activated carbons. This implies their superior appropriateness in describing the adsorption behavior.

### 3.4 Optimization studies

ANOVA results and fitness values for Cu, MB and 2,4-D adsorption are listed in Table 9. Coded second-order models showing the relationship between parameters and Cu adsorption capacity are specified in Eqs. (Eq. 18), (Eq. 19)) for KOSAC and PAC, respectively. In all of the equations, *A* stands for the temperature and *B* stands for the initial contaminant concentration.

(Eq. 18) $$q_{KOSAC}=141.99-28.37*A+23.97*B-8.73*AB+0.9113*A^{2}-17.19*B^{2}$$

(Eq. 19) $$q_{PAC}=160.72-28.27*A+26.06*B-8.06*AB-1.01*A^{2}-19.14*B^{2}$$

**Table 9:** ANOVA results for Cu, MB and 2,4-D adsorption.

Cu adsorption							
KOSAC				PAC			
Source	Degree of freedom	F-value	P-value	Source	Degree of freedom	F-value	P-value
**Model**	5	153.62	<0.0001	**Model**	5	107.96	<0.0001
A	1	136.50	0.0010	A	1	81.27	<0.0001
B	1	97.75	<0.0001	B	1	69.36	<0.0001
AB	1	17.47	0.0013	AB	1	8.93	0.0113
A^2^	1	0.0683	0.7983	A^2^	1	0.0499	0.8270
B^2^	1	82.96	<0.0001	B^2^	1	61.67	<0.0001
**Residual**	12			**Residual**	12		
**Cor Total**	17			**Cor Total**	17		
**Fit Statistics**				**Fit Statistics**			
Std. Dev.	6.98			Std. Dev.	9.01		
Mean	103.79			Mean	117.25		
C.V. %	6.72			C.V. %	7.68		
R^2^	0.9846			R^2^	0.9783		
Adjusted R^2^	0.9782			Adjusted R^2^	0.9682		
Predicted R^2^	0.9541			Predicted R^2^	0.9315		
**MB adsorption**							
**ZOSAC**				**PAC**			
**Source**	**Degree of freedom**	**F-value**	**P-value**	**Source**	**Degree of freedom**	**F-value**	**P-value**
**Model**	5	1064.58	<0.0001	**Model**	5	440.98	<0.0001
A	1	120.07	<0.0001	A	1	20.69	0.0007
B	1	891.69	<0.0001	B	1	461.91	<0.0001
AB	1	37.30	<0.0001	AB	1	3.32	0.0934
A^2^	1	0.0990	0.7584	A^2^	1	0.2127	0.6529
B^2^	1	624.05	<0.0001	B^2^	1	193.81	<0.0001
**Residual**	12			**Residual**	12		
**Cor Total**	17			**Cor Total**	17		
**Fit Statistics**				**Fit Statistics**			
Std. Dev.	9.77			Std. Dev.	22.58		
Mean	320.93			Mean	457.12		
C.V. %	3.04			C.V. %	4.94		
R^2^	0.9978			R^2^	0.9946		
Adjusted R^2^	0.9968			Adjusted R^2^	0.9923		
Predicted R^2^	0.9926			Predicted R^2^	0.9859		
**2,4-D adsorption**							
**ZOSAC**				**PAC**			
**Source**	**Degree of freedom**	**F-value**	**P-value**	**Source**	**Degree of freedom**	**F-value**	**P-value**
**Model**	5	540.56	<0.0001	**Model**	5	541.22	<0.0001
A	1	105.71	<0.0001	A	1	193.38	<0.0001
B	1	904.21	<0.0001	B	1	677.23	<0.0001
AB	1	11.92	0.0048	AB	1	59.83	<0.0001
A^2^	1	0.8267	0.3811	A^2^	1	0.4352	0.5219
B^2^	1	56.41	<0.0001	B^2^	1	135.73	<0.0001
**Residual**	12			**Residual**	12		
**Cor Total**	17			**Cor Total**	17		
**Fit Statistics**				**Fit Statistics**			
Std. Dev.	8.14			Std. Dev.	12.69		
Mean	163.67			Mean	277.72		
C.V. %	4.97			C.V. %	4.57		
R^2^	0.9956			R^2^	0.9956		
Adjusted R^2^	0.9937			Adjusted R^2^	0.9937		
Predicted R^2^	0.9886			Predicted R^2^	0.9899		

F-values of 153.62 and 107.96 for KOSAC and PAC, respectively, show that the model is significant. Terms with a p-value less than 0.0500 in the model equation are significant for the model. It is seen that the terms A, B, AB and B^2^ are significant for the Cu adsorption model. The high correlation coefficient represents the suitability of the model. For KOSAC it is 0.9846 and for PAC it is 0.9783.

Coded second-order models showing the relationship between parameters and MB adsorption capacity are specified in Eqs. (Eq. 20), (Eq. 21)) for ZOSAC and PAC, respectively.

(Eq. 20) $$q_{ZOSAC}=475.08+37.28*A+101.39*B+17.87*AB+1.54*A^{2}-66.05*B^{2}$$

(Eq. 21) $$q_{PAC}=685.17+35.75*A+168.63*B+12.32*AB-5.21*A^{2}-85.05*B^{2}$$

F-values of 1064.58 and 440.98 for ZOSAC and PAC, respectively, show that the model is significant. In MB adsorption, the terms A, B, AB and B^2^ for ZOSAC and A, B and B^2^ for PAC are seen to be significant. The high correlation coefficient represents the suitability of the model. For ZOSAC it is 0.9978 and for PAC it is 0.9946.

Coded second-order models showing the relationship between parameters and 2,4-D adsorption capacity are specified in Eqs. (Eq. 22), (Eq. 23)) for ZOSAC and PAC, respectively. A stands for the temperature and B stands for the initial contaminant concentration.

(Eq. 22) $$q_{\text{ZOSAC}}=243.81-29.15*A+85.09*B-8.42*\text{AB}-3.70*A^{2}-16.55*B^{2}$$

(Eq. 23) $$q_{\text{PAC}}=403.57-61.43*A+114.73*B-29.39*\text{AB}+4.18*A^{2}-39.99*B^{2}$$

F-values of 540.56 and 541.22 for ZOSAC and PAC, respectively, show that the model is significant. It can be seen that the terms A, B, AB and B^2^ are significant for ZOSAC and PAC in 2,4-D adsorption. A high correlation coefficient represents the suitability of the model. For ZOSAC it is 0.9956 and for PAC it is 0.9956.

**Fig. 11:**
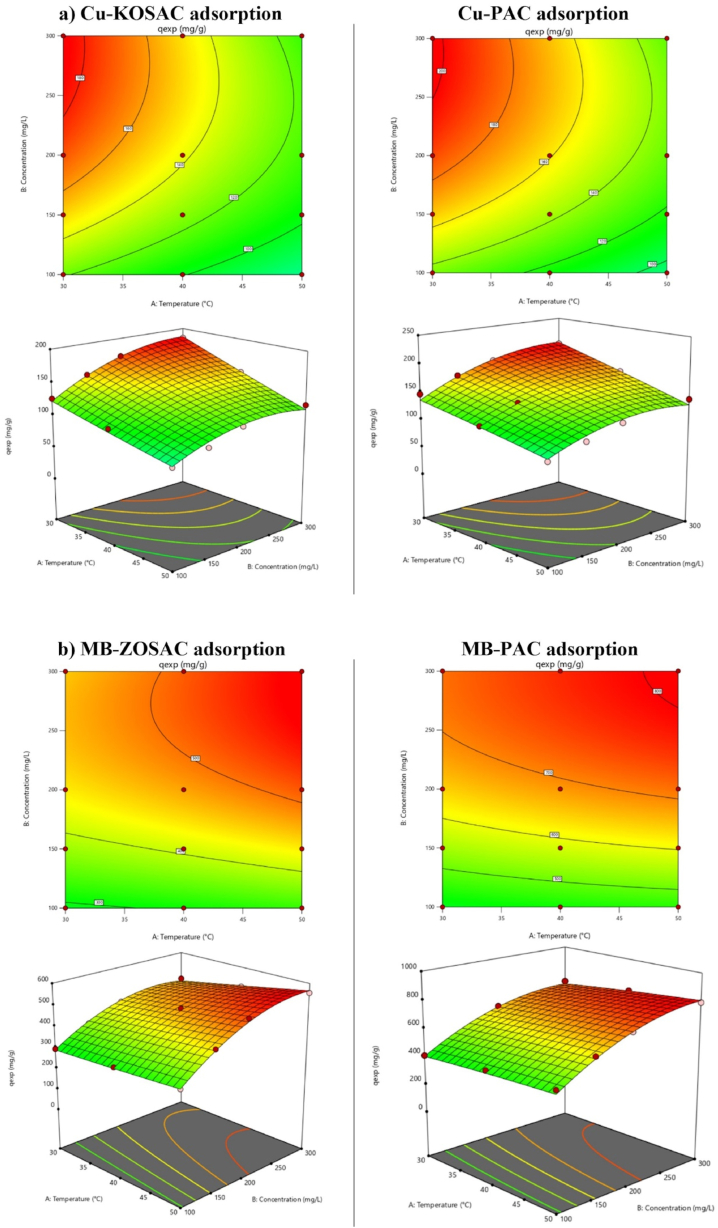

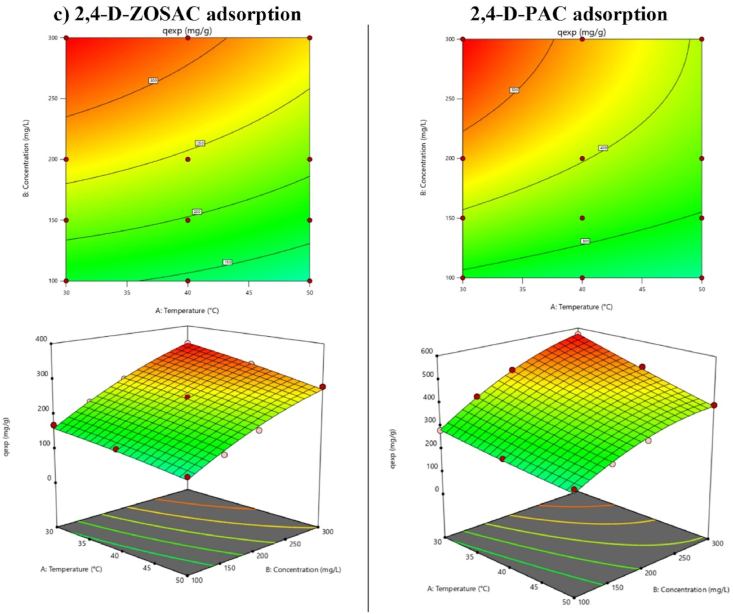
2D and 3D surface and contour graphs for **a)** Cu adsorption, **b)** MB adsorption, and **c)** 2,4-D adsorption.

The 2D and 3D surface and contour graphs for Cu, MB and 2,4-D adsorption are given in Fig. 11. The optimum experimental conditions for Cu adsorption were calculated as 30.34 °C and 297.65 mg L^−1^ for KOSAC and PAC. The optimum experimental conditions for MB adsorption were calculated as 48.62 °C and 269.37 mg L^−1^ for ZOSAC, and 48.45 °C and 282.34 mg L^−1^ for PAC. The optimum experimental conditions for 2,4-D adsorption were calculated as 30.31 °C and 299.02 mg L^−1^ for ZOSAC, and 30.06 °C and 298.14 mg L^−1^ for PAC.

The maximum adsorption capacities reached with activated or non-activated waste-based activated carbon in the literature in comparison to the maxima of this study are given in Table 10. For Cu removal, KOH-impregnated activated carbon showed an outstanding performance among other Cu adsorption studies. It can be said that the MB adsorption capacity of ZnCl_2_-impregnated activated carbon was quite acceptable. It was observed that the adsorption capacity obtained with the ZnCl_2_-impregnated activated carbon in the sorption of 2,4-D is considerably high and superior to other adsorbents. Hence, the removal efficiencies of adsorbents obtained from olive stone in this study, which is an abundantly available agricultural by-product in the Aegean Region of Turkey, showed that the produced activated carbons can be considered to be superior in water purification applications.

**Table 10:** Adsorption studies with waste-based activated carbon in the literature.

Raw Material	Activator	Pollutant	Maximum adsorption capacity (mg g^−1^)	Reference
Papaya seeds	–	Cu	17.3	[48]
Walnut shell	–	Cu	14.5	[49]
Plum kernel	H_3_PO_4_	Cu	48.3	[50]
Ginkgo leaf	–	Cu	59.9	[51]
Macroalgae	–	Cu	98.6	[52]
Olive stone	KOH	Cu	184.7	This study
Soybean waste	Silica	Methylene Blue	24.1	[30]
Sucrose	KOH	Methylene Blue	704.2	[45]
Waste paper	ZnCl_2_	Methylene Blue	1657	[32]
Olive stone	ZnCl_2_	Methylene Blue	557.5	This study
Pulp ash	–	2,4-D	7.1	[53]
Cornstalk	K_2_CO_3_	2,4-D	8.5	[24]
Rice husk	–	2,4-D	27.2	[54]
Sesame	ZnCl_2_	2,4-D	36.2	[42]
Carboneous materials	–	2,4-D	89.5	[55]
*Physalis peruviana*	H_2_SO_4_	2,4-D	320.0	[28]
Olive stone	ZnCl_2_	2,4-D	344.8	This study

## 4 Conclusions

Turkey's significant position as the fourth largest producer of olives and olive oil globally underscores the abundance of olive by-products within its agricultural landscape. With approximately 228 tons of olives accounting for 8.21 % of total production, the harvesting and processing of olives yield a substantial volume of by-products, notably olive stones and pomace. For every 1 decare of olive trees, approximately 2500 kg of olives are harvested, resulting in 500 kg of olive oil and 2000 kg of pomace, and this pomace contains 400 kg of olive stones, 40 kg of pomace oil, and 260 kg of pomace dry residue along with 1300 kg of wastewater. These olive stones and pomace dry wastes are generally used as fuel; however, instead of using this potentially high-potential waste as fuel, valuable products can be developed such as effective, low-cost, and sustainable adsorbent materials to be used for treating water resources polluted by organic and inorganic contaminants.

In this study, H_3_PO_4_, KOH, and ZnCl_2_ impregnated activated carbons were evaluated in terms of their adsorption performances across a variety of contaminants (Cu, MB and 2,4-D) and compared with commercial activated carbons. This study presents a transformative approach by harnessing the latent value of olive stones through the production of activated carbons. By impregnating olive stones with various activating agents, it unveils a pathway towards creating effective, low-cost, and sustainable adsorbent materials for water treatment applications. This represents a paradigm shift from conventional waste management practices towards value-added utilization, aligning with principles of circular economy and resource efficiency.

The findings elucidated the influence of parameters such as initial pollutant concentration, temperature, pH and adsorbent amount on adsorption capacities. For all the pollutants, as the initial concentration was increased, the adsorption capacities also increased. The highest adsorption capacities were reached at pH 5, 10, and 2 for Cu, MB and 2,4-D. Cu removal increased from 27 % to 52 % for KOSAC, MB removal increased from 39 % to 65 % for ZOSAC and 2,4-D removal increased from 33 % to 99 % for ZOSAC at different adsorbent amounts. As a result of experiments carried out at three different temperatures, it was observed that Cu and 2,4-D adsorption were exothermic, whereas MB adsorption was endothermic. Notably, the observed exothermic and endothermic nature of adsorption processes for different pollutants underscores the nuanced interplay between physicochemical factors and adsorbent characteristics.

Furthermore, adsorption mechanisms for each contaminant were investigated providing valuable insights into the underlying processes. It was found that the PFO kinetic model and the Langmuir isotherm model were more suitable for Cu adsorption, whereas the PFO and PSO kinetic models and the Redlich-Peterson isotherm models were more prominent for MB and 2,4-D adsorption. Besides, the optimum temperatures and the maximum adsorption capacities achieved were 30.34 °C and 297.65 mg L^−1^ for KOSAC-Cu, 48.62 °C and 269.37 mg L^−1^ for ZOSAC-MB and 30.31 °C, and 299.02 mg L^−1^ for ZOSAC-2,4-D interactions.

In essence, this study transcended mere scientific inquiry, embodying a holistic methodology that encompasses diverse pollutants, activating agents, performance metrics, and environmental considerations. By repurposing olive by-product into functional adsorbent material, the study not only addressed water pollution challenges but also contributed to the broader goals of sustainability and environmental stewardship. In doing so, it exemplifies a proactive approach towards leveraging agricultural waste streams for the betterment of both ecosystems and human well-being.

## Data availability statement

No data associated with this study has been deposited into a publicly available repository. However, additional raw data can be made available on request.

## CRediT authorship contribution statement

**Duygu Ova Ozcan:** Writing – review & editing, Writing – original draft, Visualization, Validation, Software, Resources, Project administration, Methodology, Investigation, Funding acquisition, Formal analysis, Conceptualization. **Mert Can Hendekci̇:** Data curation. **Bikem Ovez:** Supervision.

## Declaration of competing interest

The authors declare the following financial interests/personal relationships which may be considered as potential competing interests: Duygu Ova Ozcan reports financial support was provided by 10.13039/501100003010The Scientific Research Foundation of Ege University. If there are other authors, they declare that they have no known competing financial interests or personal relationships that could have appeared to influence the work reported in this paper.
